# In Vitro and In Silico Studies of Antimicrobial, and Antioxidant Activities of Chemically Characterized Essential Oil of *Artemisia flahaultii* L. (Asteraceae)

**DOI:** 10.3390/life13030779

**Published:** 2023-03-13

**Authors:** Khalid Chebbac, Zineb Benziane Ouaritini, Abdelfattah El Moussaoui, Mohamed Chebaibi, Ahmad Mohammad Salamatullah, Soufyane Lafraxo, Mohammed Bourhia, John P. Giesy, Mourad A. M. Aboul-Soud, Raja Guemmouh

**Affiliations:** 1 Laboratory of Biotechnology Conservation and Valorisation of Natural Resources, Faculty of Sciences Dhar El Mahraz, Sidi Mohamed Ben Abdellah University, Fez 30000, Morocco; 2 Laboratory of Natural Substances, Pharmacology, Environment, Modeling, Health and Quality of Life, Faculty of Sciences, Sidi Mohamed Ben Abdellah University, Fez 30000, Morocco; 3 Laboratory of Biotechnology, Environment, Agri-Food and Health, Faculty of Sciences Dhar El Mahraz, Sidi Mohamed Ben Abdellah University, Fez 30000, Morocco; 4 Biomedical and Translational Research Laboratory, Faculty of Medicine and Pharmacy of the Fez, Sidi Mohamed Ben Abdellah University, Fez 30000, Morocco; 5 Department of Food Science & Nutrition, College of Food and Agricultural Sciences, King Saud University, P.O. Box 2460, Riyadh 11451, Saudi Arabia; 6 Laboratory of Chemistry and Biochemistry, Faculty of Medicine and Pharmacy, Ibn Zohr University, Laayoune 70000, Morocco; 7 Toxicology Centre, University of Saskatchewan, Saskatoon, SK S7N 5B3, Canada; 8 Department of Veterinary Biomedical Sciences, University of Saskatchewan, Saskatoon, SK S7N 5B4, Canada; 9 Department of Clinical Laboratory Sciences, College of Applied Medical Sciences, King Saud University, P.O. Box 10219, Riyadh 11433, Saudi Arabia

**Keywords:** free radicals, antibacterial, antifungal, GC/MS, structure–activity modeling, natural products

## Abstract

The present study investigated the antioxidant and antimicrobial activities as well as characterized the chemical composition of the essential oils (EO) isolated from *Artemisia flahaultii* (EOF). EOF was extracted using hydro-distillation, and the chemical composition of EOF was ascertained by gas chromatography coupled with mass spectrometry (GC/MS). To assess antioxidant capacity, three tests were used: the 2,2-diphenyl-1-picrylhydrazil (DPPH), the total antioxidant capacity (TAC) and the ferric-reducing antioxidant power (FRAP) test. The antimicrobial activity of EOF was investigated using the diffusion assay and minimal inhibitory concentration assays (MICs). By use of in silico structure–activity simulations, the inhibitory potency against nicotinamide adenine dinucleotide phosphate (NADPH), physicochemical characters, pharmaco-centric properties and absorption, distribution, metabolism, excretion (ADME) characteristics of EOF were determined. GC/MS analysis reveals 25 components majorly composed of D-Limonene (22.09%) followed by β-pinene (15.22%), *O*-cymene (11.72%), β-vinylnaphthalene (10.47%) and benzene 2,4-pentadiynyl (9.04%). The capacity of DPPH scavenging by EOF scored an IC50 of 16.00 ± 0.20 µg/mL. TAC revealed that the examined oils contained considerable amounts of antioxidants, which were determined to be 1094.190 ± 31.515 mg ascorbic acid equivalents (AAE)/g EO. Results of the FRAP method showed that EOF exhibited activity with EC50 = 6.20 ± 0.60 µg/mL. Values for minimal inhibitory concentration (MIC) against certain clinically important pathogenic bacteria demonstrate EOF’s potent antibacterial activity. MIC values of 1.34, 1.79, and 4.47 μg/mL against *E. coli*, *B. subtilis* and *S. aureus* were observed respectively. EOF exhibited significant antifungal activities against two stains of fungi: *F. oxysporum* and *C. albicans*, with values of 10.70 and 2.23 μg/mL, respectively. Of the total, 25 essential oils were identified. 2,4-Di-tert-butylphenol and capillin were the most active molecules against NADPH. The ADME prediction revealed that *EOF* was characterized by useful physicochemical characteristics and pharmaco-centric properties. The findings of this study show that the EOF can be used as an alternative to treat microbial resistance. Based on the in silico studies, EOF can be used as an “eco-friendly” NADPH inhibitor.

## 1. Introduction

Due to the need to protect agricultural crops and from spoiling before sale, uses of synthetic insecticides and fungicides are increasing. Due to the toxicological risks posed by pesticides, their uses are of increasing concern. Moreover, antimicrobial drugs are undeniably one of the most crucial therapeutic discoveries of the last two centuries [1,2]. Nonetheless, a dramatic increase in bacterial resistance to antimicrobial drugs has been seen, resulting in frequent antibiotic use and insufficient control of diseases [3]. In an effort to discover new plant-based molecules with biological and antioxidant activity, researchers are assessing plant-based products, such as essential oils (EO) and extracts, as potential natural substitutes for conventional produced chemicals. Natural substances contain a variety of chemical molecules, including peptides, terpenes, polyphenols and alkaloids among others, with very diverse physicochemical properties and a wide variety of biological activities, which include antitumor, antiviral, antimicrobial, antioxidant and various therapeutic uses [4,5,6,7]. With more than 300 species, the genus *Artemisia*, in the daisy family, is widespread throughout the world [8]. Several species in this genus are known by their English common names, such as sagebrush, mugwort, and wormwood. Moroccan researchers have studied the chemical compositions of various parts of these plants and have discovered multiple classes of chemicals with various biological activities [9,10,11]. Among these plants, *Artemisia flahaultii* in the Asteraceae family has recently been characterized. *A. flahaultii* grows wild at altitudes of 2200 to 2800 m on southern slopes of mountains in the BouNacer region of eastern Morocco [12,13]. *A*. *flahaultii* has been designated as a rare and endemic, indigenous species of Morocco by the International Union for Conservation of Nature in North Africa (IUCN). *A. flahaultii* forms stand in association with other species such as *Bupleurum spinosa* and *Juniperus communis L* [13]. To the best of our knowledge, this is the first publication on the chemical composition and assessment of activities of the EO of *A. flahaultii* (EOF).

## 2. Materials and Methods

### 2.1. Reagents and Chemicals Used

2,2-Diphenylpicrylhydrazyl radical (DPPH), potassium ferricyanide (K3Fe(CN)6), iron III chloride (FeCL3), ascorbic acid, gallic acid, quercetin, ammonium molybdate, and other culture media and common synthetic antibiotics were purchased from COGELAB (Fes, Morocco).

### 2.2. Selection and Identification of Plant Material 

**Figure 1:**
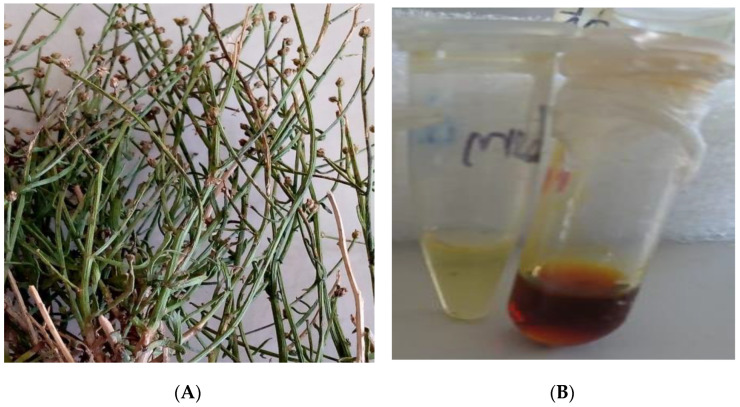
Morphology of aerial parts of *A. flahaultii* (**A**) and essential oil (dark color) (**B**).

*A. flahaultii* was collected at the end of October 2021 from the southern slope Mountain of Bou-Nacer in eastern Morocco (latitude: 33.90059736; longitude: −3.90059736; altitude: 2350 m). The botanist Amina Bari identified the spacemen and deposited a sample in the herbarium of the University of Science of Fez, Sidi Mohamed Ben Abdellah Dhar El-mahraz, Morocco (accession number: AFB001J180921). The parts of the leaves of the plant (Figure 1) were extracted after being allowed to dry for 15 days at room temperature in the shade.

### 2.3. Extraction of EOF

EOF was extracted by use of a Clevenger hydro-distillation apparatus. First, 750 mL of distilled water was added to 100 g of the aerial section, which had been coarsely chopped, and the mixture was allowed to boil for 2 h and 30 min. A temperature of 4 °C was used to store the derived EOF. Based on the dry weight of the plant components, the yield was determined and reported as a percentage (%). 

### 2.4. Analysis of the Chemical Composition of EOF by GC/MS 

#### Gas Chromatography—Mass Spectrometry (GC/MS) Analysis

After diluting the extracted oil in hexane (10 to 100 dilution), 0.001 mL was used to identify and quantify compounds by chromatographic analysis through the use of a gas chromatograph (GC) (GCMS-TQ8040 NX (Shimadzu brand)) equipped with a polar capillary column (RTxi-5 Sil MS-30 m × 0.25 mm ID × 0.25 m). The oven temperature program was set to 50 °C for 2 min (Ramp 1: 5 °C/min to 160 °C for 2 min, Ramp 2:5 °C/min to 280 °C for 2 min). The analysis time was 50 min with a 1 mL/min flow rate of nitrogen carrier gas (N2). The injector and detector were set to 250 °C and 280 °C, respectively. The sample volume injected was 1 µL; the ionization energy was 70 eV; the ionization mode was ionization; the ion source temperature was 200 °C; the scan mass range was *m/z* 40–650; and the interface line temperature was 280 °C. Constituents of EOF were identified by comparing Kovat indices of EOs, which was calculated based on the retention times of a series of linear alkanes (C4–C29), comparing with those of the reference products. In addition, Kovat indices of essential oils were compared with those of known chemical constituents, and mass spectra were compared with those gathered in a library of EOs (NIST-MS Search Version 2.0) [14].

### 2.5. In Vitro Antioxidant Activity of EO

#### 2.5.1. 2,2-Diphenyl-1-picrylhydrazyl Test (DPPH) 

Before it was carried out, small modifications were made to the previous methods developed for the DPPH test [15]. One milliliter of a freshly made 0.005% DPPH solution in methanol was mixed with 0.1 mL of various quantities of the essential oils. This sample was substituted for methanol to create the blank. The absorbance at 517 nm was measured using a Shimadzu 160-UV spectrophotometer after each mixture had been shaken and incubated at room temperature in the dark for 35 min. As positive controls, ascorbic acid, gallic acid (also known as 3,4,5-trihydroxybenzoic acid) with the formula C_6_H_2_(OH)_3_CO_2_H and quercetin were employed. The percentage of DPPH free radical inhibition was calculated (Equation (1)).

IP (%) = (A0 − A/A0) ∗ 100 (1)

where IP: Inhibition %. A0: Absorbance of the control. A: Absorbance of *EOF*.

#### 2.5.2. Ferric-Reducing Power Test (FRAP)

The FRAP test was performed according to a previously reported method [15]. In summary, 50 µL of *EOF* was mixed with 200 µL of phosphate buffer solution (200 mM-PH = 6.6) and 200 µL of potassium ferricyanide [K3Fe(CN)6] (1%). Then, the whole blend was incubated for 25 min at 52 °C in a water bath after shaking. The sample was mixed with 200 µL of trichloroacetic acid (10% TCA); 120 µL of 0.1% FeCl3 and 600 µL of distilled water are included to prepare the mixture for measurement. Absorbance was determined at 700 nm against a control (which contains 50 µL of methanol in place of *EOF*). Results were expressed as EC-50 effective concentration (a concentration equal to half (0.5) the absorbance). The graph was used to compute the median effective concentration (EC-50). 

#### 2.5.3. Total Antioxidant Capacity Test (TAC)

The TAC test was based on conversion of Mo (VI) to Mo (V) and production of a green Mo (V) phosphate complex at an acidic pH [16]. After adding 25 L of EO to 1 mL of the reagent solution (consisting of 28 mM sodium phosphate, 4 mM ammonium molybdate, and 0.6 M sulfuric acid), the mixture was heated to 95 °C for 1 h and 20 min before being allowed to cool to room temperature. With a control containing 25 μL of methanol in place of the test oil, optical density was measured at 695 nm in a spectrophotometer [4]. Ascorbic acid, gallic acid, and quercetin were used as standards, and the TAC was expressed as milligrams of ascorbic acid equivalence per gram of EO (mg AAE/g EO). No fewer than three measurements were taken for each test solution.

### 2.6. Antimicrobial Activity

#### 2.6.1. Culture Medium

For the disk diffusion method, to determine the antimicrobial potency of EOF, bacteria were grown on Muller–Hinton (MHA) agar, while yeast were grown on Pepton Glucose Yeast (YPG). The broths utilized in the microdilution method were Muller–Hinton MHB broth for bacteria and (YPG) broth for yeast. At 120 °C for 25 min, all media were autoclaved [16].

#### 2.6.2. Strains Tested and Standardization of the Inoculum

The antimicrobial activity of EOF was tested on five strains: Gram-negative bacteria including *Escherichia coli* (K12), two Gram-positive bacteria *Bacillus subtilis* (DSM6633) and *Staphylococcus aureus* (CECT976), and two fungal species (*Fusarium oxysporum* (LBEAH/FS/17) and *Candida albicans* (ATCC10231)). All strains investigated were obtained from lung, urinary tract, and surgical site infections in clinical patients in the intensive care unit of the University Hospital Complex, Fez, Morocco. Selected microbial strains were grown in tubes containing 9 mL of Mueller–Hinton broth before being incubated at 37 °C for 18 to 24 h (MHB). A drop of the culture was plated onto Petri dishes containing nutrient agar using a platinum loop, and it was then incubated once again for 18 to 24 h at 37 °C. The bacterial suspension (inoculum) was prepared from the pure cultures as follows: Three to five identical colonies were scraped with a platinum loop and discharged into 5 mL of sterile physiological water with 0.9% NaCl in a sterile hemolysis tube. A 0.5 McFarland adjustment was made to turbidity. The amount of CFU per milliliter in bacterial suspensions ranged between 1–2 × 10^8^ and 1–5 × 10^5^ in fungal suspensions. The McFarland standard was prepared with a mixture of 99.5 mL of 0.36 N sulfuric acid solution (H_2_SO_4_) and 0.5 mL of 0.048 M dehydrated barium chloride solution (BaCl_2_*2H_2_O). According to McFarland, a concentration of 10^7^ to 10^8^ CFU/mL was determined by adjusting the optical density of the bacterial suspension to be between 0.08 and 0.1 nm. The microdilution method was used to determine the minimum inhibitory concentrations (MICs) against the tested bacterial strains [17].

#### 2.6.3. Disc Diffusion Method

To determine the zone of inhibition, the disk diffusion method was used. This technique is used to evaluate the susceptibility of microorganisms to an antimicrobial agent. After 30 min of drying, sterilized 6 mm diameter discs (grade 2) were inoculated and impregnated with 10 µL of the test substances, which were placed on the agar surfaces of the Petri dish. Effects of EOF were compared to effects of Ampicilin 0.5 mg/mL, kanamycin 0.05 mg/mL, and fluconazol 5 mg/mL. Dishes were incubated at 37 °C for bacteria. After incubation, by measuring the zones of growth inhibition in millimeters, the antibacterial potencies were determined [18,19].

#### 2.6.4. Determination of the Minimum Inhibitory Concentration (MIC) 

The microdilution technique was used to determine the minimum inhibitory concentration (MIC) in 96-well microplates [20]. Because of the immiscibility of the EO with water and therefore with the culture medium, the emulsification was carried out with a 0.15% agar solution in order to favor the germ/compound contact. Ampicilin, kanamycin and fluconazol were diluted in distilled water, and microbial suspensions at 0.5 McFarland were diluted in 0.9% physiological water (10^−6^ dilution is used for bacteria and 10^−4^ is used for fungi). With the exception of the first well, which was the negative control, 50 μL of the culture medium (MH for bacteria and YPG for fungi) and 50 μL of the diluted essential oil were then added to each of the microplate’s wells, which were the positive growth, control. Following that, microdilutions were performed by moving 50 μL from the first well to the second well and so on. Dilutions of 1/2 were made sequentially with a range from 870 to 1.4 μg/mL. Finally, wells were inoculated by placing 50 μL of the microbial solution that had been tested for turbidity. The microplate was incubated for 18 h for bacteria, 48 h for *C. albicans*, and 7 days for fungi (*Fusarium oxysporum*) at 37 °C and 30 °C, respectively. To read the results, each well received 15 μL of a 0.015% resazurin aqueous solution to visualize microbial growth. The MIC was defined as the lowest concentration that did not create a pink color [21,22].

### 2.7. Molecular Docking

All constituents identified in EOF from *A. flahaultii* L. were downloaded from the PubChem database in SDF format. Then, they were prepared by use of the LigPrep tool in the Maestro 11.5 version of the Schrödinger Software program using the OPLS3 force field. A maximum of 32 stereoisomers were produced for each ligand after ionization states at pH 7.0 ± 2.0. Using the PDB ID 2CDU, three-dimensional crystal structures of NADPH oxidase in PDB format were downloaded from the protein data bank. The Protein Preparation Wizard of Schrödinger-Maestro v11.5 was used to prepare and refine the structure. The minimization of structure was carried out using an OPLS3 force field. The receptor grid had the following coordinates: X = 19.9, Y = −6.4 and Z = −0.9 when the volumetric spacing performed is 20 × 20 × 20. SP flexible ligand docking was carried out in Glide of Schrödinger-Maestro v 11.5.

### 2.8. ADME Prediction

The Qikprop function in the Maestro 11.5 edition of the Schrödinger Software was used to determine the absorption, metabolism, distribution and excretion properties. The prediction was based on the physicochemical characteristics and pharmacokinetic properties such as molecular weight, a hydrogen bond acceptor and donor, total solvent surface area, the blood–brain partition coefficient, the octanol/water partition coefficient, and aqueous solubility of EO from *A. flahaultii* L.

### 2.9. Statistical Analysis

Results of this study are presented using means as well as standard deviations of triplicate assays. The Shapiro–Wilks test and the Levene’s test were used, respectively, to verify normal distribution and homogeneity of variance. ANOVA and Tukey’s HSD post hoc analysis of variance were used to manage multiple comparisons. A significant difference was defined when *p* value less than 0.05. 

## 3. Results

### 3.1. Yield and Chemical Composition

**Figure 2:**
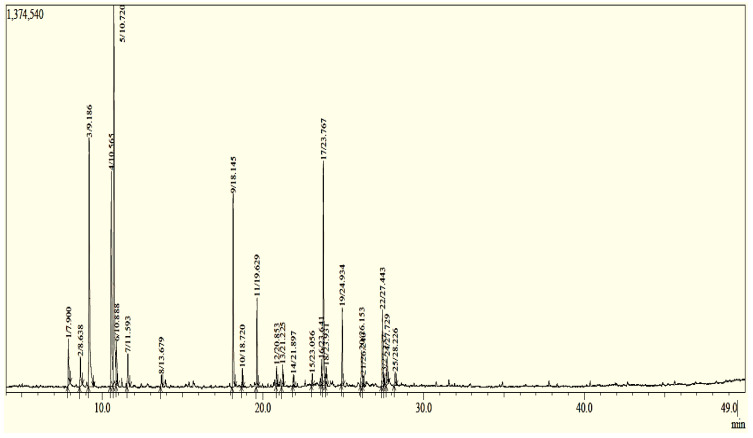
Chromatographic profile of EOF profiled by GC/MS.

**Figure 3:**
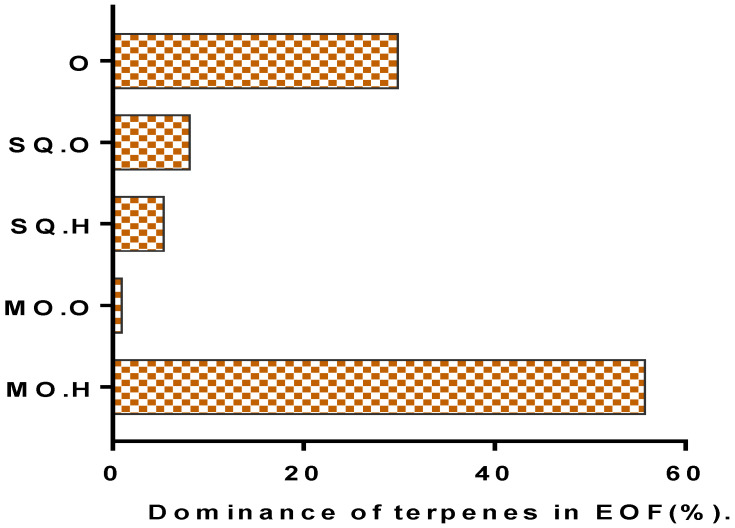
The proportions of terpenic compounds in the EOF. MO.H: Monoterpene hydrocarbons; MO.O: Oxygenated monoterpenes; SQ.H: Sesquiterpene hydrocarbons; SQ.O: Oxygenated sesquiterpenes; O: Other compounds.

Essential oil is a mobile dark red liquid with a specific smell; their oil yield was 0.46% for dry and airy raw materials (Table 1). The 25 chemicals identified in EOF accounted for 99.98% of the overall EO. The EOF is composed mainly of mono-terpenoids (56.68%) and sesqui-terpenoids (13.43%). The hydrocarbon compounds represent 61.08% of the EOF and the oxygenated compounds represent only 9.03% (Figure 2 and Figure 3 and Table 2). The main components consisted of D-limonene (22.09%), β-pinene (15.22%), *O*-cymene (11.72%), β-vinylnaphthalene (10.7%), and benzene, 2,4-pentadiynyl (9%) according to GC/MS analysis (Figure 2, Table 3). 

### 3.2. Scavenger Effect of 2,2-Diphenyl-1-picrylhydrazil (DPPH)

**Figure 4:**
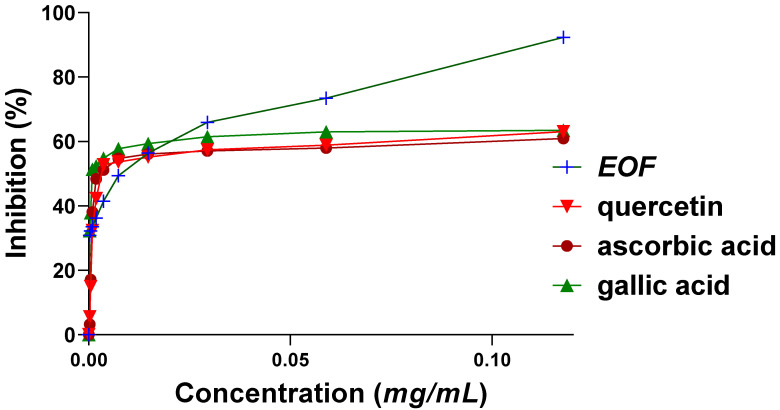
Variation in percentage of inhibition of DPPH according to the concentrations of EOF and standards (quercetin, ascorbic acid, and gallic acid).

When antioxidant activity was evaluated by the DPPH test, the IC50 values of EO were 16 ± 0.2 µg/mL greater than the IC50 values of positive controls (quercetin and ascorbic acid), which were 45 ± 0.4 µg/mL and 44 ± 0.9 µg/mL, respectively, and less than that of gallic acid, which was IC50 = 2 ± 0.4 µg/mL. Results of an ANOVA analysis showed no significant difference between the IC50 value of EOF and those of the standards (*p* > 0.05) (Figure 4).

### 3.3. Investigating the Total Antioxidant Capacity (TAC)

**Figure 5:**
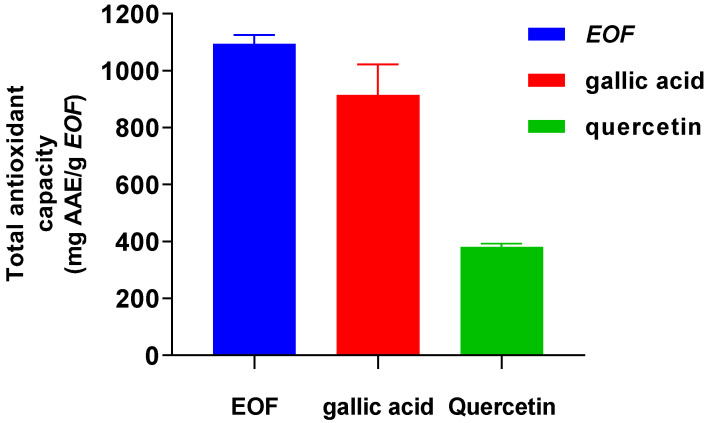
Total antioxidant capacity of EOF and standards (gallic acid or quercetin).

The phosphorus-molybdenum process, which is essentially based on the reduction of Mo (VI) to Mo (V) in the presence of an antioxidant, was used to calculate the TAC of the analyzed EOF and standard antioxidants, gallic acid or quercetin [23]. The TAC of EOF was significantly increased more than gallic acid or quercetin with values of 1094.19 ± 31.51; 914.92 ± 107.24 and 380.76 ± 11.28 mg ascorbic acid equivalents (AAE)/g EOF, respectively (Figure 5).

### 3.4. Ferric-Reducing Antioxidant Power Assay (FRAP)

**Figure 6:**
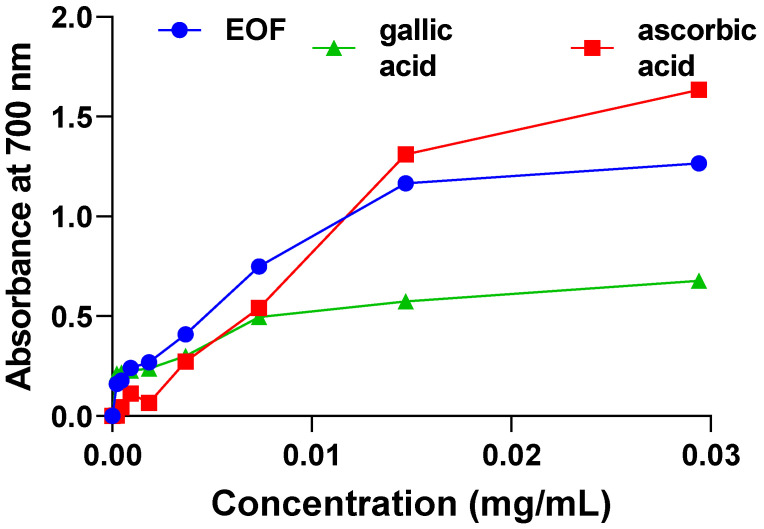
Reducing power of EOF, compared to that of ascorbic acid or gallic acid, used as references.

The capability of reduction plays an important role in the presence of antioxidants that work by donating hydrogen atoms to free radicals to break their bonds [24]. The ferric-reducing power of the studied EO was evaluated using the FRAP test to investigate the development of the reducing power (absorbance) of different concentrations used of EOF in comparison with ascorbic acid and gallic acid (Figure 6). EOF has a more effective ferric-reducing power activity with a value of EC50 = 6.2 ± 0.6 µg/mL than that of the synthetic antioxidants gallic acid or ascorbic acid with values of EC50 = 15.0 ± 1.5 µg/mL and EC50 = 7.3 ± 0.5 µg/mL, respectively.

### 3.5. Antimicrobial Activity of EOF

**Figure 7:**
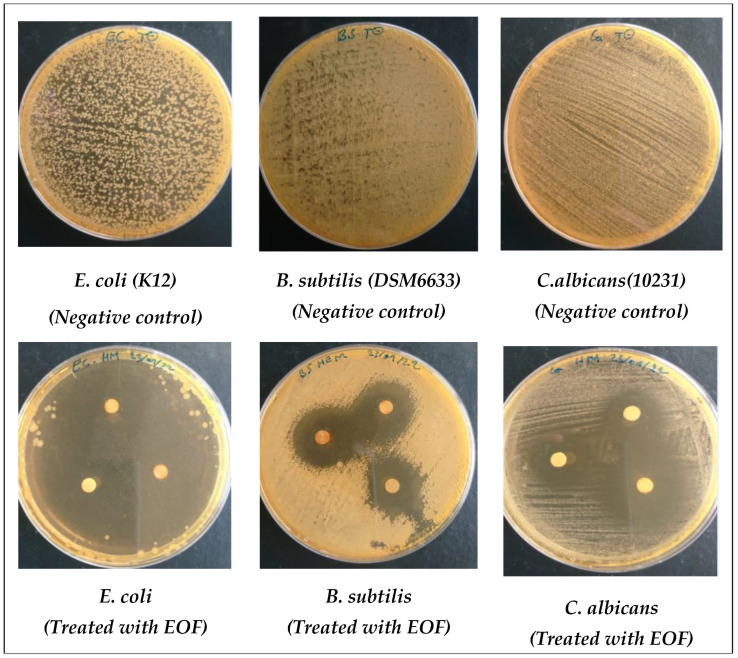
Photographs displaying effects of EOF on the tested bacteria and fungi.

In this work, when the susceptibility of microbes to the effects of EOF was tested against three bacterial strains, including *E. coli*, *B. subtilis*, and *S. aureus*, and two fungal strains, *F. oxysporum* and *C. albicans*, as the infectious, multi-resistant, and frequently contaminating germs [25], measured using the diameter of the zone of inhibition (Table 3) and MIC bioassays (Table 4), Gram (−) bacteria (*E.coli*) were sensitive to effects of EOF. Results obtained showed that Gram-negative bacteria (*E.coli*) are very sensitive to our EOF, since the maximum inhibition zone and MIC value were in the order of 68.6 ± 1.2 mm and 1.3 ± 00.00 μg/mL, respectively, toward this oil (Table 3 and Table 4), which was followed by fungal species (*F. oxysporum* and *C. albicans*), which were completely inhibited by an inhibition zone of 48.3 ± 1.5 mm with an MIC value of 10.7 ± 2.5 μg/mL for *F. oxysporum* and 35 ± 0.8 mm with 2.2 ± 0.9 mm for *C. albicans*. In contrast, Gram-positive bacteria were less sensitive to effects of EOF since the zone of inhibition and MIC value were in the range of 31 ± 1 mm, 18.3 ± 1.5 mm and 1.8 ± 0.9 and 4.5 ± 1.8 µg/mL for *B. subtilis* and *S. aureus*, respectively (Figure 7). All strains tested were resistant to antibiotics, with the exception of *E. coli* (Gram −), which was sensitive to Ampicilin with a zone of inhibition of 10.7 ± 0.6 mm but resistant to kanamycin. Regardless of the dose tested, EOF was more effective with MIC = 2.2 ± 0.9 μg/mL at inhibiting fungal growth than the positive control fluconazol with MIC = 3.8 ± 0.2 μg/mL.

### 3.6. Molecular Docking

All constituents of EOs identified in *Artemisia flahaultii* L. inhibited NADPH activity with a glide g score between −5.896 and −2.207 kcal/mol (Table 5). 2,4-Di-tert-butylphenol, isospathulenol, capillin and β-vinylnaphthalene are the most active molecules in the active site of NADPH with docking g scores of −5.896, −5.485, −5.436 and −5.387 kcal/mol, respectively.

**Figure 8:**
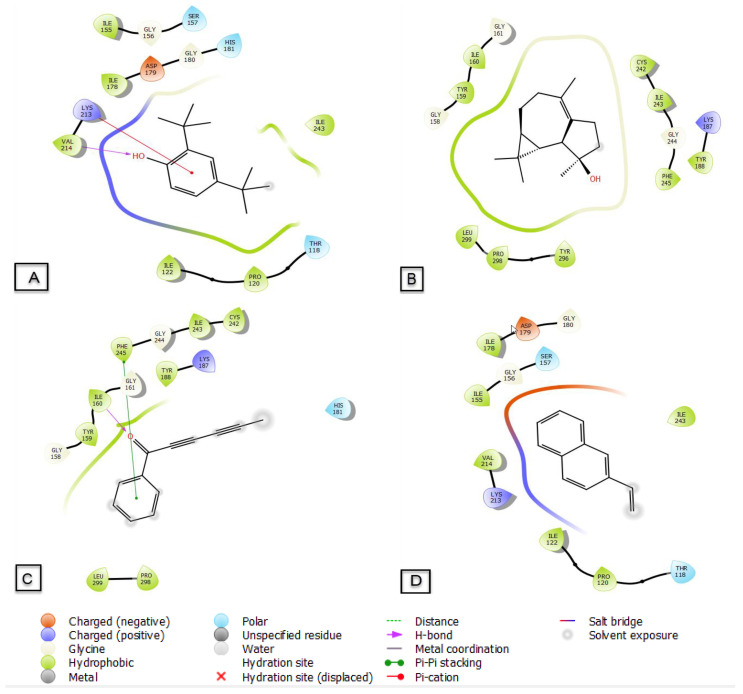
Two-dimensional (2D) diagrams of ligands interactions with the active site of NADPH. (**A**) 2,4-Di-tert-butylphenol, (**B**) isospathulenol, (**C**) capillin, (**D**) β-vinylnaphthalene.

**Figure 9:**
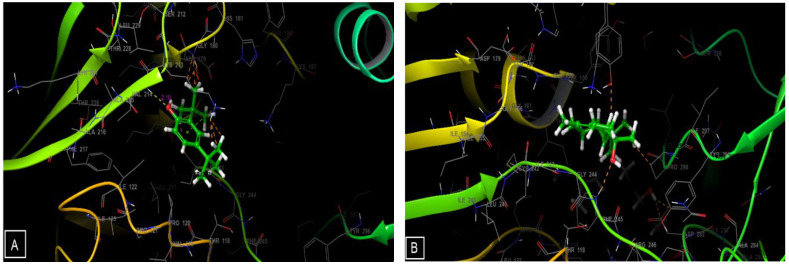

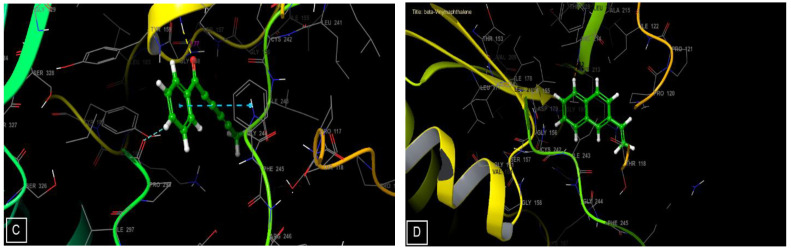
Three-dimensional (3D) diagrams of ligands interactions with the active site of NADPH. (**A**) 2,4-Di-tert-butylphenol, (**B**) isospathulenol, (**C**) capillin, (**D**) β-vinylnaphthalene.

The types and numbers of bonds between EOF and the active site of NADPH are shown (Figure 8 and Figure 9). 2,4-Di-tert-butylphenol establishes a single hydrogen bond with the VAL-214 residue and another Pi-cation type bond with the LYS-213 residue, while capillin forms a hydrogen bond with the ILE-160 residue and a Pi–Pi stacking bond with the PHE-245 residue.

## 4. Discussion

The present study investigated antioxidant and antimicrobial activities as well as characterized the chemical composition of the essential oils (EO) isolated from *Artemisia flahaultii* (EOF). The rate of EOF yield is relatively the same compared to yields obtained by other researchers for the genus Artemisia: *A. mesatlantica* endemic to Morocco and *A. annua* from France lead to a yield of approximately 0.5% [26,27]. However, the yield of the essential oil used in this study was less than certain oils of *Artemisia annua*, Iranniene, *Artemisia compestris*, and Tunisian *Artemisia herba alba*, whose yields were about 1.2% [28,29,30].

The results of phytochemical analysis obtained in this work showed that EOF had greater amounts of bioactive compounds, which are known to have effects on pathogens causing diseases in humans. Alternatively, researchers studying the EO of two species of Artemisia, Iranian *A. annua* and Canadian *A. ludoviciana*, found that sesquiterpene and oxygenated monoterpene chemicals made up 83.7% and 12.5%, respectively, of Iranian *A. annua’s* EO [21], while these chemical groups represented 48.8% and 17.8% of the EO of the Canadian plant *A. ludoviciana* [29] and 63.28% and 7.61% of the EO for *A. negrei* [4]. A similar composition of Tunisian *A. campestris* EO and endemic Moroccan *A. ifranensis* EO have been reported in previous research [31,32,33].

The DPPH of EOF with an IC50 value of 16 ± 0.2 µg/mL was significantly more potent than that found for essential oils derived from *A. herba alba* of southwestern Tunisian origin, which had an IC50 of 50.0 μg/mL [23]. EOF contains greater amounts of the major constituent compound D-limonene than do other foods, so it might be a promising source of antioxidants and radical scavengers. The antioxidant capacity of EOF was attributable primarily to D-limonene. Absolute amounts of antioxidant capacity depend on the method chosen, the concentrations used, and the phytochemical properties of the antioxidants sought [34,35].

Based on current findings, given that an EO is a complex mixture of several hundred chemicals, it is challenging to identify those constituents responsible for the antioxidant potencies of EOs. It is also challenging to elucidate mechanisms of action of EOs because of their complexity. In fact, results of research have demonstrated that the antioxidant activities of EOs can be superior to those of the essential element of substances studied independently [36,37]. This predominance of the activity of EO mixtures relative to that of individual major components confirms the synergistic effect that minor components can have on activities of EOs [38,39]. In the case of EOF, the antioxidant activity of the mixture was probably related to the essential elements, which are mainly monoterpenes, which are all known to possess antioxidant properties. As a general rule, EOs rich in oxygenated hydrocarbon compounds have greater antiradical activities than do those with oxygenated terpenes [40], but the absolute antioxidant activities differed depending on tests employed [41].

In the work presented here, *E. coli* appears to be more sensitive to EOs. Gram-negative bacteria have been shown to be more sensitive to EOs than Gram-positive bacteria. The presence of hydrocarbon monoterpenes discovered in EOF, including D-limonene, β-pinene and *O*-cymene, have been shown to have pharmacological actions in mixtures with other oxygenated monoterpenes, which might explain the antibacterial effects of EOF. These results are consistent with those of other researchers, which demonstrated that potencies of EOs toward bacteria and fungi are a function of the phytochemical families present in the EOs [42]. The lipophilic properties of EOs, which enable entry into and inhibit or kill bacterial cells, can explain the antibacterial properties of EOs. According to this theory, hydrocarbons in EOs preferentially bind to biological membranes, disrupting membrane permeability and ultimately causing microbes to quickly die [38]. The phytochemicals, D-limonene, β-pinene, *O*-cymene, and β-vinylnaphthalene, in EOs might also work synergistically rather than individually [43]. In order to have antibacterial actions, antimicrobial drugs interact with certain biochemical targets in microorganisms. Antimicrobial medications are frequently rendered ineffective in bacteria due to a variety of resistance mechanisms, which eventually allow the emergence of bacterial strains resistant to the compounds being studied. However, due to their lipophilic nature, EOs can pass through cell walls and cytoplasmic membranes, killing bacteria by upsetting structures of polysaccharides, fatty acids, and phospholipids [38]. Based on the results presented here, EOF had essentially the same potency against both Gram-positive and Gram-negative bacteria and thus has potential as a potent broad-spectrum antibacterial agent to suppress pathogenic and multidrug-resistant strains. Previously published results of studies on mechanism of action of EOs on fungi showed that more effective EOs are richer in thymol and that p-cymene kills cells by damaging membranes [44,45]. Thymol and *p*-cymene oil have been shown previously to have fungicidal effects on species of *Candida. sp* by inflicting indirect damage to the cytoplasm and membranes of bacteria [44].

2,4-di-tert-butylphenol is an essential oil found in 169 species including bacteria, fungi, plants and animals. It exhibits a significant antioxidant activity according to several scientific studies [46,47,48]. Capillin exhibits remarkable antioxidant activity and is another component of the essential oil, and it has been found in the genus Artemisia [49,50,51]. Its bioavailability is dependent on the relative rates of absorption, distribution metabolism and excretion (ADME) of the active compound; this bioavailability is directly affected by the physicochemical properties of the compound [52]. Meanwhile, the ADME prediction makes it possible to determine the psychochemical characters and the pharmacokinetic properties of the compounds identified in the essential oils of the plant. All EOs observed in *Artemisia flahaultii* L. have an acceptable molecular masses (<500 µ) except for cyclooctasiloxane and hexadecamethyl, which have a molecular mass greater than 500 µ. Concerning donors and acceptors of hydrogen bonds, all essential oils presented acceptable values (≤5 and ≤10 respectively) (Table 6). The total surface area accessible to the solvent directly influences the oral bioavailability of drug molecules [53]. All EOs showed acceptable values (range of 00–1000). Blood–brain partition coefficients were indicated by the capacity of the molecule to cross the blood–brain barrier. However, the acceptable range for the predicted blood–brain partition coefficient is −3 to −1.2 [54]. All the molecules of our study showed insignificant values (outside the interval −1.2 −3) with the exception of cinnamic acid methyl ester and benzaldehyde, which presented acceptable values (−2.47 and −1.109 respectively). The predicted percentage of oral absorption for all molecules studied was predicted to be essentially 100%.

## 5. Conclusions

This work highlights the chemical composition, antioxidant, and antimicrobial potentials of essential oil of *Artemisia flahaultii* L. Results showed that this oil possessed promising antioxidant and antimicrobial potentials vs. drug resistant microbes. Further works on toxicity in non-target organisms are needed prior to any applications of this oil as medicines. 

## Figures and Tables

**Table 1:** Physical characteristics of the essential oils of *A. flahaultii* L.

	Flowering Stage
Essential oil yields (%)	0.47
Color	Brown-yellow
Aspect	Oily

**Table 2:** Phytochemical components identified in EOF by GC/MS.

Peak	R.T (min)	Name	Area (%)	R.I		Chemical Classes
				Lit	Obs	
1	7.900	α-Pinene	2.18	932	928	MO.H
2	8.638	Benzaldehyde	1.65	952	952	O
3	9.186	β-pinène	15.22	979	973	MO.H
4	10.565	*O*-Cymene	11.72	1026	1022	MO.H
5	10.720	D-Limonene	22.09	1024	1018	MO.H
6	10.888	β-Ocimene, (E)-	2.18	1044	1048	MO.H
7	11.593	γ-Terpinene	1.77	1059	1058	MO.H
8	13.679	Neo-allo-ocimene	0.62	1140	1139	MO.H
9	18.145	Benzene, 2,4-pentadiynyl-	9.04	1212	1206	O
10	18.720	Cyclohexasiloxane, dodecamethyl-	0.71	1243	1240	O
11	19.629	Δ-Elemene	3.82	1340	1335	SQ.H
12	20.853	Cinnamic acid, methyl ester	0.96	1452	1447	MO.O
13	21.225	Methyleugenol	0.93	1376	1371	O
14	21.897	Caryophyllene	0.52	1464	1464	SQ.H
15	23.056	Cycloheptasiloxane, tetradecamethyl-	0.43	1446	1447	O
16	23.641	β-Selinene	1.02	1490	1490	SQ.H
17	23.767	β-Vinylnaphthalene	10.47	1446	1439	O
18	23.931	2,4-Di-tert-butylphenol	1.07	1557	1555	O
19	24.934	Elemicin	3.59	1555	1550	O
20	26.153	Caryophyllene oxide	2.52	1570	1567	SQ.O
21	26.240	Spathulenol	0.53	1578	1579	SQ.O
22	27.443	Isospathulenol	4.25	1625	1626	SQ.O
23	27.537	Cyclooctasiloxane, hexadecamethyl-	0.38	1554	1554	O
24	27.729	Capillin	1.56	1637	1641	O
25	28.226	Ledol	0.77	1602	1600	SQ.O

R.T: Retention time (minutes); Obs: Observed; Lit: Literature; R.I: Retention index; MO.H: Monoterpene hydrocarbons; MO.O: Oxygenated monoterpenes; SQ.H: Sesquiterpene hydrocarbons; SQ.O: Oxygenated sesquiterpenes; O: Other compounds.

**Table 3:** EOF-induced inhibition zones and controls (kanamycin, ampicillin and fluconazole) vs. fungal species and bacterial strains (mm).

Compound	Gram (−) Bacteria	Gram (+) Bacteria		Fungal Strains	
	*E. coli*	*B. subtilis*	*S. aureus*	*F. oxysporum*	*C. albican*
EOF	68.6 ± 1.1 ^a^	31.0 ± 1.0 ^b^	18.3 ± 1.5 ^c^	48.3 ± 1.5 ^d^	35.0 ± 0.8 ^b^
Ampicillin	10.6 ± 0.5 ^a^	-	-	NT	NT
Kanamycin	-	-	-	NT	NT
Fluconazole	NT	NT	NT	22.3 ±1.1 ^a^	32.6 ±2.5 ^b^

Row values with different letters are not significantly different (*n* = 3, ANOVA, Tukey’s HSD, *p*-value less than 0.05 considered to be significant).

**Table 4:** Minimum inhibitory concentration induced by EOF and positive controls μg/mL for ampicillin, kanamycin and fluconazole vs. fungal species and bacterial strains.

Compound	Gram (−) Bacteria	Gram (+) Bacteria		Fungal Strains	
	*E. coli*	*B. subtilis*	*S. aureus*	*F. oxysporum*	*C. albicans*
EOF	1.3 ± 0.0 ^a^	1.7± 0.9 ^a^	4.4 ± 1.8 ^b^	10.7 ± 2.5 ^c^	2.23 ± 0.9 ^a^
Ampicillin	8.4 ± 0.8 ^a^	-	-	NT	NT
Kanamycin	1.8 ± 0.5 ^a^	2.5 ± 0.4 ^b^	2.2 ± 0.0 ^b^	NT	NT
Fluconazole	NT	NT	NT	2.62 ± 0.0 ^a^	3.82 ± 0.2 ^a^

Row values with different letters are not significantly different (*n* = 3, ANOVA, Tukey’s HSD, *p*-value less than 0.05 considered to be significant).

**Table 5:** Docking results with EOF in active site of NADPH (PDB: 2CDU).

	Glide G Score (Kcal/mol)	Glide E-Model (Kcal/mol)	Glide Energy (Kcal/mol)
2,4-Di-tert-butylphenol	−5.896	−27.646	−21.898
Isospathulenol	−5.485	−28.834	−21.318
Capillin	−5.436	−34.526	−25.391
Beta-Vinylnaphthalene	−5.387	−29.17	−21.924
*O*-Cymene	−5.344	−23.239	−17.415
Elemicin	−4.961	−38.333	−29.49
γ-terpinene	−4.906	−24.602	−18.813
Benzaldehyde	−4.65	−24.218	−18.527
Spathulenol	−4.508	−24.42	−19.062
β-Selinene	−4.492	−16.319	−13.148
Cinnamic acid, methyl ester	−4.471	−32.984	−25.141
Benzene, 2,4-pentadiynyl-	−4.43	−28.246	−22.272
Caryophyllene	−4.343	−11.897	−11.633
Caryophyllene oxide	−4.144	−19.805	−18.17
α-Pinene	−4.09	−13.27	−10.049
D-Limonene	−4.02	−16.575	−14.119
Methyleugenol	−3.896	−28.648	−23.187
Ledol	−3.663	−15.216	−13.583
Neo-allo-ocimene	−3.272	−19.08	−16.142
δ-Elemene	−3.108	−11.06	−11.65
β-Pinene	−2.819	−9.574	−8.187
Cycloheptasiloxane, tetradecamethyl	−2.582	−32.429	−28.287
(E)-β-Ocimene−1	−2.207	−17.529	−16.436

**Table 6:** ADME properties of constituents of essential oils (EOF) extracted from *Artemisia flahaultii* L.

Compound Name	MM ^a^	Donors HB ^b^	Acceptors HB ^c^	SASA ^d^	QPlogPo/w ^e^	QPlogBB ^f^	QPlogS ^g^	%Human Oral Absorption ^h^
Ledol	222.4	1	0.75	460.5	4.0	−4.15	0.33	100
Capillin	168.2	0	2	441.4	2.8	−3.14	−0.10	100
Cyclooctasiloxane Hexadecamethyl	593.2	0	1.6	947.9	11.8	−10.7	−0.48	100
Isospathulenol	220.3	1	0.75	454.2	3.7	−4.03	0.21	100
Spathulenol	220.3	1	0.75	464.7	3.9	−4.23	0.25	100
Caryophyllene oxide	220.3	0	2	431.0	2.5	−4.33	0.09	100
Elemicin	208.2	0	2.25	465.8	2.9	−3.93	−0.08	100
2,4-Di-tert-butylphenol	206.3	1	0.75	467.5	3.8	−3.91	0.12	100
β-Vinylnaphthalene	154.2	0	0	386.4	4.1	−4.3	0.321	100
β-Selinene	204.4	0	0	464.7	5.3	−6.34	1.01	100
Methyleugenol	178.2	0	1.5	426.9	2.9	−3.77	0.10	100
Cinnamic acid methyl ester	162.2	0	2	414.7	2.4	−2.47	−0.20	100
δ-Elemene	204.4	0	0	472.9	5.5	−6.56	1.01	100
Benzene, 2,4-pentadiynyl-	140.2	0.5	0	397.3	4.0	−3.41	0.36	100
Neo-allo-ocimene	136.2	0	0	425.9	4.6	−4.80	0.93	100
GAMMA-TERPINENE	136.2	0	0	394.3	4.1	−4.16	0.85	100
(E)-β-Ocimene	136.2	0	0	412.2	4.4	−4.69	0.88	100
D-Limonene	136.2	0	0	386.5	4.0	−4.00	0.83	100
*O*-Cymene	134.2	0	0	373.7	3.7	−4.04	0.64	100
β-Pinene	136.2	0	0	361.5	3.5	−4.02	0.86	100
Benzaldehyde	106.1	0	2	300.0	1.5	−1.10	−0.08	94
α-Pinene	136.2	0	0	367.3	3.6	−4.02	0.87	100

^a^ mass of molecules (acceptable range: 500 amµ). ^b^ Donor of hydrogen bonds (acceptable range: ≤5). ^c^ Acceptor of hydrogen bonds (acceptable range: ≤10). ^d^ Total solvent accessible surface area using a probe with a 1.4 radius (acceptable range: 300–1000 radius). ^e^ Predicted octanol/water partition coefficient (acceptable range: −2–6.5). ^f^ Predicted blood–brain partition coefficient (acceptable range: −3–1.2). ^g^ Predicted aqueous solubility, S in mol/dm−3 (acceptable range: −6.5–0.5). ^h^ Predicted human oral absorption on 0 to 100% scale (<25% is poor and >80% is high).

## Data Availability

Included within the article.
